# Maize Introgression Library Provides Evidence for the Involvement of *liguleless1* in Resistance to Northern Leaf Blight

**DOI:** 10.1534/g3.120.401500

**Published:** 2020-08-14

**Authors:** Judith M. Kolkman, Josh Strable, Kate Harline, Dallas E. Kroon, Tyr Wiesner-Hanks, Peter J. Bradbury, Rebecca J. Nelson

**Affiliations:** * School of Integrative Plant Science, Plant Pathology and Plant-Microbe Biology Section, Cornell University, Ithaca, NY 14853; † School of Integrative Plant Science, Plant Biology Section, Cornell University, Ithaca, NY 14853; ‡ US Department of Agriculture, Agricultural Research Service, Ithaca, NY 14853; § School of Integrative Plant Science, Plant Breeding and Genetics Section, Cornell University, Ithaca, NY 14853

**Keywords:** *Zea mays*, *Setosphaeria turcica*, near-isogenic lines, *liguleless1*, introgression library, Multiparent Advanced Generation Inter-Cross (MAGIC) multiparental populations MPP

## Abstract

Plant disease resistance is largely governed by complex genetic architecture. In maize, few disease resistance loci have been characterized. Near-isogenic lines are a powerful genetic tool to dissect quantitative trait loci. We analyzed an introgression library of maize (*Zea mays*) near-isogenic lines, termed a nested near-isogenic line library for resistance to northern leaf blight caused by the fungal pathogen *Setosphaeria turcica*. The population was comprised of 412 BC_5_F_4_ near-isogenic lines that originated from 18 diverse donor parents and a common recurrent parent, B73. Single nucleotide polymorphisms identified through genotyping by sequencing were used to define introgressions and for association analysis. Near-isogenic lines that conferred resistance and susceptibility to northern leaf blight were comprised of introgressions that overlapped known northern leaf blight quantitative trait loci. Genome-wide association analysis and stepwise regression further resolved five quantitative trait loci regions, and implicated several candidate genes, including *Liguleless1*, a key determinant of leaf architecture in cereals. Two independently-derived mutant alleles of *liguleless1* inoculated with *S. turcica* showed enhanced susceptibility to northern leaf blight. In the maize nested association mapping population, leaf angle was positively correlated with resistance to northern leaf blight in five recombinant inbred line populations, and negatively correlated with northern leaf blight in four recombinant inbred line populations. This study demonstrates the power of an introgression library combined with high density marker coverage to resolve quantitative trait loci. Furthermore, the role of *liguleless1* in leaf architecture and in resistance to northern leaf blight has important applications in crop improvement.

Quantitative trait locus (QTL) mapping has been used to dissect the genetic architecture of many important agronomic traits in crop plants, including disease resistance. Resistance to disease in plants has largely focused on qualitative disease resistance loci, which can have large effect but can become easily overcome by the pathogen. Disease resistance QTL that show a quantitative level of resistance usually have small to moderate effects on disease phenotypes, and the underlying polymoprhisms are not well known. Quantitative disease resistance loci have typically been identified through associations between DNA markers and disease phenotypes through linkage mapping or genome -wide association studies (GWAS). The maize nested mapping association mapping (NAM) population (Yu *et al.* 2008), a multi-parent recombinant inbred line (RIL) population, has increased the resolution of QTL for many traits, including several disease traits (Kump *et al.* 2011, Poland *et al.* 2011, Benson *et al.* 2015, Li *et al.* 2018). A population of near-isogenic lines (NILs) was developed in parallel with the NAM (Gandhi *et al.* 2001), and has been used for detailed analysis of selected QTL (Ding *et al.* 2017).

Comparing a NIL to the corresponding recurrent parent allows the effects of specific chromosomal segment(s) to be assessed. While there are logistical advantages to use a population with a uniform genetic background, there is no theoretical advantage in the power to detect QTL using NILs *vs.* RILs (Kaeppler 1997). Studies using Arabidopsis have suggested that NILs may be able to detect smaller allelic effects in NILs relative to RILs (Keurentjes *et al.* 2007; Marchadier *et al.* 2019), however using the same populations sizes, NILs offer less resolution (Keurentjes *et al.* 2007). The use of NILs can permit diverse alleles at a locus of interest to be compared in a common and adapted genetic background, potentially allowing the identification of novel sources of variation for breeding programs (Young *et al.* 1988; Bernacchi *et al.* 1998). QTL can be resolved to detect candidate or causal genes through breakpoint analysis by further backcrossing of selected NILs for fine mapping (Eshed and Zamir 1995; Monforte and Tanksley 2000; Jeuken and Lindhout 2004). Utilizing RILs typically allows QTL mapping at low resolution, and fine mapping with NILs can also be limited in regions of low recombination (*e.g.*, Jamann *et al.* 2014). NILs have been used in genetic studies of quantitative disease resistance in maize for QTL discovery (Chung *et al.* 2010; Lopez-Zuniga *et al.* 2019, Morales *et al.* 2020), for phenotypic analysis (Chung *et al.* 2011; Jamann *et al.* 2014; Zhang *et al.* 2017; Morales *et al.* 2020) and for identifying the genes underlying QTL (Yang *et al.* 2017).

Resistance to northern leaf blight (NLB), caused by the fungal pathogen *Setosphaeria turcica*, is inherited both quantitatively and qualitatively. Many QTL and several race specific genes for NLB have been identified in biparental mapping populations (Welz and Geiger 2000; Wisser *et al.* 2006; Galiano-Carneiro and Miedaner 2017). The genetic architecture of NLB resistance was further analyzed in the NAM population (Poland *et al.* 2011), which consists of 5,000 RILs derived from crosses between B73 and 25 diverse founder lines (Buckler *et al.* 2009, McMullen *et al.* 2009, Yu *et al.* 2008). Using the NAM population, 23 and 49 NLB QTL were identified using 1,106 SNPs (Poland *et al.* 2011) and 7,386 SNPs (Li *et al.* 2018), respectively. Several NILs carrying major and minor NLB loci have been developed and characterized for resistance to diseases (Chung *et al.* 2010; Chung *et al.* 2011; Balint-Kurti *et al.* 2010; Belcher *et al.* 2012; Lopez-Zuniga *et al.* 2019; Morales *et al.* 2020). Many NLB QTL, however, remain to be characterized in detail.

This paper presents the characterization of a set of ∼450 maize NILs derived from a subset of the maize NAM founder (donor) lines backcrossed to the recurrent B73 inbred line. We used genotyping by sequencing (GBS; Elshire *et al.* 2011) to provide high density SNP coverage across the collection of NILs, termed here as a ‘nested NIL’ (nNIL) library. The nNIL library was evaluated for resistance to NLB over three years of inoculated field trials. Because the donor lines are genetically diverse, both the ancient and recent recombination events within the uniform genetic background were harnessed using genome wide association studies (GWAS) to aid in resolving QTL for resistance to NLB. While not all QTL were represented by a sufficient diversity of donor lines or NIL coverage to allow extensive QTL dissection through association analysis, five regions of the genome harboring NLB QTL were targeted with significant resolution. In one such region, the *liguleless1* (*lg1*) gene was implicated through GWAS. Inoculation of lines carrying mutant alleles of *lg1* showed that *lg1* importantly plays a previously undescribed role in resistance to NLB, suggesting that *lg1*, in addition to regulating leaf architecture traits, may control factors that influence disease resistance.

## Materials and Methods

### NILs

A total of 453 NILs derived from a subset of the NAM founders (Yu *et al.* 2008) were developed by, and obtained from Syngenta Agrochemical Company (Gandhi *et al.* 2001) from a larger set of approximately 1500 NILs. The NILs were created by crossing the NAM founders with B73, followed by five backcross generations and three generations of self-pollination to create BC_5_F_3_ NILs. The NILs were requested with the intention of finding introgressions covering chromosomal segments containing QTL for resistance to NLB, gray leaf spot (GLS) and aflatoxin accumulation, and were chosen by Syngenta based on a proprietary linkage map. The NILs contained introgressions from 18 donor lines, of which, 13, 3 and 2 donors were derived from the tropical, mixed and non-stiff stalk (NSS) sub-populations in maize, respectively (Figure S1). Ten seeds from each of the selfed BC_5_F_3_ NILs (*i.e.*, S4’s) were grown and self-pollinated at the Cornell University Musgrave Research Farm in Aurora, NY in 2010 to produce BC_5_F_4_ seed.

### NIL genotyping and analysis

The NIL library was genotyped by sequencing (Elshire *et al.* 2011). Four seeds of each line were planted in a 96 cell insert pack and grown under greenhouse conditions. Fresh tissue was harvested from up to 4 seedlings per NIL and DNA was extracted using the Qiagen Plant DNA extraction kit (Qiagen, Germantown, MD, USA). DNA was quantified and checked for quality using the restriction enzyme *EcoR*I. Approximately 30 to 50 ng of DNA was used for 384-plex DNA sequencing at the Institute for Genomic Diversity at Cornell University, Ithaca, NY, USA. SNPs were called using the TASSEL5 GBSv1 production pipeline with the ZeaGBSv2.7 TagsOnPhysicalMap (TOPM) file, as described in Glaubitz *et al.* (2014). Imputation was performed with TASSEL-FILLIN (Swarts *et al.* 2014), using the publicly available haplotype donors file AllZeaGBSv2.7impV5_AnonDonors8k.tar.gz (see https://www.panzea.org/genotypes). GBS data for 955,680 SNPs (B73 AGPv2) were obtained on 412 NILs of the 453 NILs (File S1). Of the 41 excluded NILs, 37 had poor or no GBS sequence data 3 had no introgressions, and 1 NIL was an exact duplicate of an existing NIL included in the analysis. Verification of the introgression donor parents and the start and end introgression sites were defined using TASSEL (Bradbury *et al.* 2007) and SNPbinner (Gonda *et al.* 2019).

### Identification of introgression

Initial graphical genotypes (Young and Tanksley 1989) were produced for each NIL using an R script that compared GBS-derived DNA sequences and represented each SNP with a vertical line. One set of graphical genotypes compared each NIL with B73. Introgressions were identified by the high density of SNPs relative to B73. In a second analysis, the NIL sequence was compared to its putative introgression donor line. If the donor was correctly identified, the second graphical genotype was the inverse of the first. If the donor was not correctly identified, the introgression would only be seen in the contrast with B73.

The donor parents were further confirmed using the imputed SNP data. For each NIL, all non-B73 SNPs were compared to each of the 18 donor lines. Using Python, this was executed at the whole-genome level, and for each of the putative introgression sites identified. If a clear best match was evident for all introgressions and the overall genome, the donor was declared. If several potential donors for a given introgression had similar match scores, the best overall genome match was used to resolve ambiguities. The sequence data were also inspected to identify heterozygous regions. The putative donor was also considered in ambiguous cases.

Introgression endpoints were determined using the Python-based program SNPbinner (https://github.com/solgenomics/SNPbinner). SNPbinner was developed for defining breakpoints in GBS data for recombinant inbred lines (Gonda *et al*. 2019); to our knowledge, this study is the first instance in which this program is being used for NILs. The GBS data were converted to “abh” SNP data in TASSEL5 (Bradbury *et al.* 2007), where ‘a’ was the B73 parent, ‘b’ was the donor parent, and “h” was a heterozygous call. The minimum introgression size was set to 1.5 Mb as a standard for each chromosome. This threshold that was well below 3.25% of the genome size that would be expected for a BC_5_ introgression (ranging from 4.9 to 9.8 Mb per chromosome), but large enough to prevent SNPbinner from breaking apart single introgressions into many small consecutive segments. When necessary, introgressions were visually inspected in TASSEL. Nineteen NILs were found to have small introgressions that were not immediately identified via SNPbinner and were identified upon visual inspection of the NIL HapMap file in TASSEL. The introgressions were visualized using a horizontal line graph in ggplot2 (R Studio) and organized to represent the tiling path across the maize genome.

### Phenotyping NILs and maize diversity panel

The 453 BC_5_F_4_ NILs were planted in randomized complete block designs with two replications per year in 2011, 2012 and 2013 at the Cornell University Musgrave Research Farm in Aurora, NY, and planted on June 3^rd^, May 27^th^ and May 17^th^, respectively. The 282 line maize diversity panel (Flint-Garcia *et al.* 2005) was grown in 2006 and 2007 in NY, and 2007 in NC, as previously reported (Wisser *et al.* 2011) with an additional environment in 2008 in NY. The diversity panel and the nNIL library were inoculated with *Setosphaeria turcica* isolate NY001 (race 1) at the 6 to 8 leaf stage in the field as previously described (Chung *et al.* 2010). Each plant received two types of inoculum in the leaf whorl at the 6 to 8 leaf stage: 500 µl of a spore suspension of approximately 4,000 spores/ml and approximately 1.3 ml sorghum kernels that had been colonized by *S. turcica*. The lines were scored on a row basis three times for DLA after flowering, at intervals of 10 to 14 days. DLA area was scored across all replications in each environment for both the NIL and diversity panel trials. Area under the disease progress curve (Jeger and Viljanen-Rollinson 2001) per day was determined from the three DLA scores (Das *et al.* 1992), and standardized (sAUDPC) to account for the duration of the scoring interval (Balint-Kurti *et al.* 2010).

Flowering time was scored as days to anthesis (DTA) after planting for both replications of the nNIL trial in 2011 and 2012, and one replication for the NILs in 2013. Average and range of DTA in the nNIL trials were converted to Growing Degree Units for comparison. DTA was measured in the 2 replications of each of the four environments of NLB trials for the 282 maize inbred diversity panel. DTA was determined as the number of days after planting when half of the plants in the row had started pollen shed. The nNIL experiment was analyzed as a randomized complete block design in a mixed model including genotype, replication nested within environment, environment and genotype*environment as random effects to create best linear unbiased prediction (BLUP) estimates for AUDPC and DTA. Variance components were derived to calculate heritability on an entry means basis, where *h^2^* = V_g_/(V_e_/rt+V_gxe_/t+V_g_), with V_g_, V_e_ and V_gxe_ representing genetic variance, experimental error and genotype x environment interaction respectively, and r and t representing number of replications and test environments, respectively (Fehr 1987).

### Analysis of the NILs for Resistance to NLB

To identify pairwise differences between NILs and the recurrent parent B73, we used a least squares model with AUDPC and DTA as fixed effects to estimate a Dunnett’s pairwise multiple comparison in JMP Pro Version 13.1.0 (SAS Institute, Inc, Cary, NC, 1989-2019). NILs with AUDPC and DTA values outside the 95% confidence interval of B73 were also identified. The introgressions of the NILs that were significantly more or less resistant to NLB than B73 were compared to prior joint linkage (JL) mapping of NLB QTL in the NAM (Poland *et al.* 2011). These QTL were mapped from germplasm of similar origin (recombinant inbred lines of the NAM), grown at the same location and inoculated with the same NLB isolate. Allelic effects of NLB QTL were estimated for the NILs and compared with the estimates based on the JL QTL in the NAM RIL populations. The QTL designations (Chung *et al.* 2010) reference to the disease, bin and donor based on the “favorable” (*i.e.*, resistance) allele (*e.g.*, qNLB2.01_B73_). If a QTL had multiple founders that contributed resistance, it was designated qNLB2.01_NAM_. A tiling path visualization was created using ggplot2 in R (R Studio), graphically representing the introgressions for each NIL.

### Association mapping in the nNIL library

In order to refine candidate regions and/or genes for resistance to NLB, we employed association analysis in the nNIL library using the sequence differences among the introgression donors, in a uniform B73 genetic background. PROC GLM was utilized in TASSEL, using the AUDPC BLUPs for NLB in the nested NIL library. The AGPv2 SNPs were filtered using a minor allele frequency (MAF) of 0.001 and minimum count of 20. Significance thresholds were determined using the Bonferroni correction factor and false discovery rate (FDR). Stepwise regression in TASSEL was used independently of the GWAS analysis, but utilizing the same SNP dataset, to identify the SNPs contributing the most toward variation in NLB, using an entry limit of 1 × 10^−5^ and an exit limit of 2 × 10^−5^. Candidate genes and annotated function for the identified SNPs were located using MaizeGDB (www.maizedgb.org; Portwood *et al.* 2019).

### Effect of liguleless1 on NLB

Two *lg1* mutant alleles were tested for resistance to NLB: the *lg1-R* allele (Moreno *et al.* 1997) in a B73 genetic background, and the *lg1-mum* allele derived from UFMu-1038042 in a W22 genetic background (Settles *et al.* 2007). The *lg1* mutant lines and corresponding B73 and W22 inbred lines were tested for resistance to NLB in two greenhouse trials, with four replications per trial and 6 to 8 plants per genotype in each replication. Plants were inoculated with a liquid spore suspension into the whorl during the late afternoon as described above. Overhead sprinklers provided a mist of water for 10 sec every 10 min for approximately 12 to 15 hr. The NLB incubation period was scored as the number of days following inoculation when a necrotic lesion was observed. Greenhouse trials were conducted during a seasonal time period that was found to mimic field inoculation results (Chung *et al.* 2010).

### NLB association with leaf angle

Leaf angle measurements for the NAM population were obtained from Panzea (www.panzea.org). Spearman’s correlations were determined using JMP Pro Version 13.1.0 between upper leaf angle BLUPs (36) and NLB AUDPC BLUPs (Poland *et al.* 2011) in each of the 26 NAM RIL populations separately.

### Genic Association to NLB in liguleless1

Regional association analysis was conducted using MLM in TASSEL with the 282 maize diversity panel based on the NLB AUDPC BLUPs estimated across four environments, with environment, replication nested within environment, and genotype x environment as random effects, and DTA as a fixed effect in BLUP estimation. The HapMapV3.2.1 LLD (high confidence) SNP dataset (panzea.org) that spanned the *lg1* (GRMZM036297) gene was used for this analysis, including 2 kb upstream and downstream of the gene. The MLM analysis used population structure and kinship as previously reported (Samayoa *et al.* 2015). Bonferroni correction and FDR were used to determine significant SNP associations (Bonferroni 1936; Benjamini and Hochberg 1995).

### Data availability

The NILs described in this manuscript are available upon request through CIMMYT. Raw resequencing data of the near-isogenic lines is deposited in the NCBI SRA (http://www.ncbi.nlm.nih.gov/sra) under accession number PRJNA640414. Supplemental information includes supplemental Tables, Figures, and a Dataset. The NIL introgressions are described in Table S1, including donor parent designation, introgression start and end points, introgression type and designation of resistance or susceptibility in comparison to B73. Phenotypic data of the NIL, including NLB AUDPC and days to anthesis are in Table S2, along with donor designation information. Tables S3 and S4 included introgression SNP matching and genomewide SNP matching data, respectively, for use in calling or confirming introgression parents. The stepwise regression statistics for the nested NIL library GWAs are located in Table S5, and Table S6 includes information on the numbers of NILs, NIL donors and NLB phenoptye for given NILs in the five GWAS/Stepwise regression regions identified in Tables 1 and S5. Python code for parent introgression and genome matching is available at (https://bitbucket.org/kharline/nelson_lab/src/master/). The NIL hapmap genotyping data (AGPv2) are available in File S1. Supplemental material available at figshare: https://doi.org/10.25387/g3.12522224.

**Table 1:** SNPs associated with resistance to northern leaf blight (NLB) in the nested near-isogenic line library were identified through genome-wide association and stepwise regression. The three most significant GWAS SNPs are listed for each of the four significant GWAS regions that exceeded a Bonferroni threshold. Across the genome, five SNPs were identified via stepwise regression as significantly contributing to resistance to NLB, including several that overlap with significant GWAS SNPs listed.

Chr	SNP (AGPv2)	*p*-value (GWAS)	*p*-value (Step Reg.)	Gene model	Function	NLB QTL
1	183,611,752	1.1857 x 10^−11^	—	AC194176.3	Low confidence gene	qNLB1.06
	191,522,802	1.3495 x 10^−10^	2.29 x 10^−15^	GRMZM2G132712	Ca^2+^ -transporting ATPase	qNLB1.06
	182,904,724	1.7486 x 10^−10^	—	GRMZM2G107162	Unknown function	qNLB1.06
8	129,860,923*^a^*	5.8631 x 10^−10^	—	GRMZM2G043954	unknown function	qNLB8.05
	121,797,691	1.3194 x 10^−9^	—	GRMZM2G366873	Auxin-responsive GH3 family protein	qNLB8.05
	120,958,608	2.2179 x 10^−9^	—	Intergenic	–	qNLB8.05
	138,561,937	5.0306 x 10^−9^	2.97 x 10^−13^	GRMZM2G136765	Protein phosphatase	qNLB8.05
5	193,652,476	4.62x10^−10^	8.11 x 10^−12^	GRMZM2G005996	mechanosensitive channel of small conductance	qNLB5.05
	192,307715	4.9454 x 10^−10^	—	GRMZM2G112830	trpp6 – trehalose-6-phosphate phosphatase6	qNLB5.05
	191,129,001	1.6531 x 10^−8^	—	GRMZM2G094768	DUF630/DUF632 domains containing protein	qNLB5.05
6	141,511,189	1.2292 x 10^−7^	—	GRMZM2G357834	wound-induced protein WI12	qNLB6.05
				GRMZM5G883407	Transposable element	
	141,836,653	1.2642 x 10^−7^	4.09 x 10^−11^	GRMZM2G023105	ENTH/VHS/GAT family protein	qNLB6.05
	134,458,026	1.4661 x 10^−7^	—	GRMZM2G055678	proline extensin-like receptor kinase 1	qNLB6.05
2	4,263,855	1.6107 x 10^−5^	5.91 x 10^−7^	GRMZM2G036297	squamosa promoter binding protein-like 8; *lg1*	qNLB2.01/2.02

a Additional SNPs at 129,860,924 and 129,860,927 were also identified with this SNP and are segregating together.

## Results

### Characterization of the NILs

**Figure 1:**
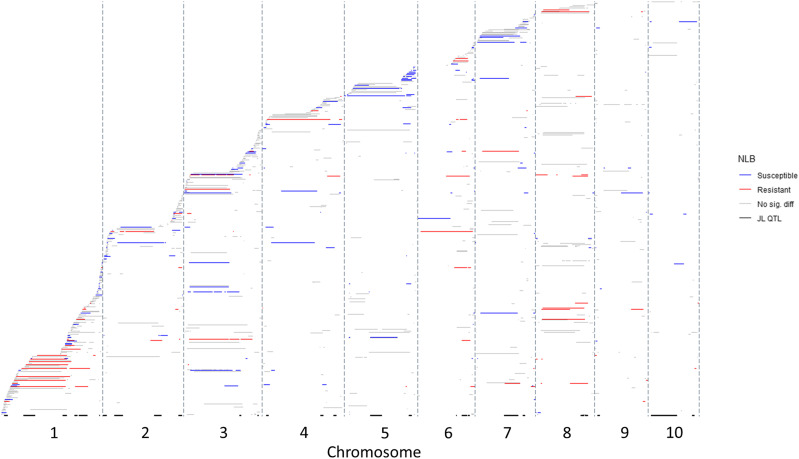
Introgressions in near-isogenic lines (NILs) across the maize genome, ordered along the y-axis based on the first introgression start site. Introgressions marked in red, blue and gray represent NILs that were more resistant, susceptible or not significantly different to northern leaf blight (NLB) in comparison to B73, respectively. Black bars along the X-axis represent the NLB quantitative trait loci mapped in the nested association mapping population (Poland *et al.* 2011). Gray vertical bars delineate the chromosomes.

We analyzed a set of NILs that were derived from crosses between 18 diverse maize inbred lines and the recurrent parent B73 (termed nNILs for the nesting of diverse haplotypes within introgressed intervals). The donor lines were founders of the widely-utilized NAM population (Figure S1; Buckler *et al.* 2009, McMullen *et al.* 2009, Yu *et al.* 2008). In total, we identified 1001 introgressions across 412 NILs, covering the entire genome (Figure 1; Table S1; File S1). There were on average 2.4 introgressions per NIL, with 29% (n = 118) of the NILs having only one introgression. The total set of introgressions summed to 24.6 Gb, with an average of 59.7 Mb per NIL, or 2.9% of the genome per NIL. The average introgression size across the genome was 24.7 Mb with a median introgression length of 9.4 Mb, and a range from 59 to 188,059 Kb. 51.6% of the introgressions were under 10 Mb. Introgressed regions spanned the genome and were not biased toward a given genomic region or introgression donor (Figure S2.). The 1001 introgressions were comprised of 620 homozygous introgressions, 209 heterozygous introgressions, and 172 introgressions that included both homozygous and heterozygous segments (Figure S3.). The 1277 introgressions included 852 segments that were homozygous and 425 segments that were heterozygous (Table S1). The introgressed segments were 81% homozygous for a total of 19,919 Mb (average = 23.4 Mb), and 19% heterozygous, totaling 4,667 Mb (average = 11.0 Mb).

We identified 247 NILs in which the introgressions matched the original putative donor parents based on the graphical genotyping (*e.g.*, Figure S4). Of the remaining lines, 136 and 17 NILs were flagged as putatively incorrect and suspect, respectively. The majority of these cases were readily resolved by comparing SNPs at the specific introgression sites, with one of the donor lines showing a much higher match than the others. In general, the total genome-wide SNP matching was consistent with the interval-specific SNP matching (Tables S2, S3 and S4). In several cases, however, there was no outstanding donor match; in these cases, inspection of the NIL HapMap file revealed heterozygous introgressions. In these cases, the interval-specific match, genome-wide match and the original putative parent identity were taken into consideration in calling the most likely donor. Of the 412 genotyped NILs, 259 (65%) of the NILs matched the original putative parent, and 141 (35%) of the NILs matched another donor parent. Twelve lines harbored small introgressions that had breakpoints that were determined visually using the HapMap file.

Across the majority of NILs, two regions of the genome harbored what appeared to be introgressions, but were more likely sequencing artifacts or polymorphisms associated with different B73 seed sources used in different research groups (Liang and Schnable, 2016). Between the regions of approximately 289.85 Mb and 293.15 Mb of chromosome 1, SNP variants appeared to produce two main haplotypes across the set of NILs. A second region with SNP variants across the entire NIL set was identified on chromosome 5, between approximately 208.16 and 211.54 Mb; this region also showed two main haplotypes. These sequence artifacts were not included in the defined introgressions.

### Phenotypic traits in the nNIL library

Phenotypic analysis of the nested NIL library, consisting of 453 NILs, was conducted in 2011-13 for days to anthesis (DTA) and three times post-flowering for diseased leaf area (DLA). The average (and range) of DLA for 2011, 2012 and 2013 was 27% (6.3–50%), 26% (5.3–52%) and 26% (6.0–54%), respectively, with B73 averaging 30% (2011), 25% (2012), and 21% (2013). Area under the disease progress curve (AUDPC) and DTA across years appeared to be normally distributed (Figure S1). Genotype and genotype-by-environment effects were significant for AUPDC, while replication and environment effects were not. Planting of 2011 trial was delayed to due excessive rainfall in May, followed by extreme drought conditions that led to the use of irrigation not only for disease establishment but for overall plant health. Heavy fall rains contributed toward a late flush of northern leaf blight. In contrast, the 2013 growing environment was conducive for an epidemic of northern leaf blight across the New York growing region (Mueller *et al.* 2016). The estimated heritability on an entry means basis was 0.74 for AUDPC and 0.36 for DTA. The average (and range) of DTA across the years 2011 - 2013 was 68.4 days (61.0-80.0 days), 76.8 days (68.5-84.5 days) and 88.0 days (81.0-103.0 days) respectively, with the average DTA of B73 being 68.4 days (2011), 76.8 days (2012) and 86.3 days (2013). The average (and range) of Growing Degree Days (GDD) across years 2011 – 2013 the three years was 1361 (1207-1590), 1529 (1351-1666) and 1570 (1450-1815), respectively, with the average GDDs of B73 being 1361 (2011), 1526 (2012), and 1692 (2013). The genotype effect was significant for DTA while the environment, replication and the genotype by environment interaction were not. In the nNIL library, there was a small but significant negative correlation between AUDPC and DTA (*R^2^*=-0.025; *P* = 0.0009; Figure S1). In contrast, there was a highly significant negative correlation between AUDPC and DTA in the 282 maize diversity panel (*R^2^* = -0.39; *P* < 0.0001; Figure S5), which may largely be due to population structure. All but the tropical subpopulations showed a significant negative correlation between DTA and AUDPC. There was less variability in NLB and DTA in the tropical subpopulation, which as a group was more resistant to NLB, and had greater DTA in comparison to other subpopulations.

Estimated allelic effects for resistance to NLB (calculated as AUDPC, based on % DLA) in the NILs ranged from -13.7 to 16.0 (mean = 0.00). In contrast, the range of allelic effects for resistance to NLB (based on % DLA) in the NAM Joint Linkage QTL (Poland *et al.* 2011) ranged from -5.6 to 5.3 (mean = 0.07). In this study, four NILs showed significantly higher levels of NLB resistance relative to B73 based on the Dunnett’s multiple test comparison. An additional 33 NILs were more resistant to NLB than B73 based on a 95% confidence interval (Table S2). The most resistant NIL had a large introgression on chromosome 6, covering a known QTL at bin 6.05 (Chung *et al.* 2011; Poland *et al.* 2011). Averaged across 3 years, this line showed a 17.5% decrease in NLB. The other three most resistant NILs based on Dunnett’s test for multiple comparison all harbored introgressions covering a large section of chromosome 1 and showed 15.6, 14.7 and 13.9% lower NLB than B73. Twenty NILs were significantly more susceptible to NLB than B73 based on the Dunnett’s multiple test comparison, with 56 additional NILs more susceptible to NLB based on a 95% confidence interval.

Thirty-four NILs had significantly higher DTA relative to B73, and three NILs had significantly lower DTA relative to B73 based on a Dunnett’s test. Five of the 34 NILs also had significantly different to DTA relative to B73 (Table S2). Three of the NILs with an effect on DTA had been identified as more susceptible to NLB (compared to B73). These included a NIL with an introgression on the end of chromosome 1; a NIL with a large introgression on chromosome 8 that overlaps with flowering time locus *Vegetative to generative transition1* (*Vgt1*; Salvi *et al.* 2007; Ducrocq *et al.* 2008) and two other introgressions; and an NLB-susceptible NIL with an introgression at 12-36 Mb on chromosome 1, and second small introgression (<2 Mb) on chromosome 7. Two NILs with significantly different DTA were more resistant to NLB. The NIL with the largest DTA harbored an introgression that spans a large area on chromosome 8 where both major flowering time (*Vgt1*) and NLB resistance *genes Helminthosporium turcicum resistance2* (*Ht2)* and *Helminthosporium turcicum resistanceN1* (*HtN)* reside (Chung *et al.* 2010; Hurni *et al.* 2015). The other NLB-resistant, high-DTA NIL had a small introgression on chromosome 1, and large introgressions on chromosomes 7 and 8 (Tables S1 and S2).

The introgression tiling path revealed several trends (Figure 1). Introgressions from any single parent were distributed across the genome (Figure S2), making breakpoint analysis from individual donors an unsuitable means to resolve QTL. As expected based on patterns of recombination (Rodgers-Melnick *et al.* 2015), smaller introgressions were identified on the ends of the chromosomes (Figure 1). Introgressions spanning centromeres tended to be large, and two regions of the genome were associated with particularly large, resistance-associated introgressions. Introgressions spanning the centromeric region of chromosome 1 (∼80 to 200 Mb) were frequently associated with resistance to NLB, perhaps reflecting the known NLB QTL in bin 1.06 (Wisser *et al.* 2006; Chung *et al.* 2011; Jamann *et al.* 2014). Introgressions on chromosome 8 also tended to be large and associated with resistance, likely reflecting the major genes and QTL that have been identified in that region (Chung *et al.* 2010; Wisser *et al.* 2006; Hurni *et al.* 2015). In contrast, introgressions on chromosome 5 were often associated with increased susceptibility to NLB compared with B73, which has a known NLB resistance QTL in bin 5.05 (Poland *et al.* 2011; Li *et al.* 2018; Wisser *et al.* 2006). Although the B73-derived resistance in bin 5.05 was displaced by introgressions that increased susceptibility in seven NILs, breakpoint analysis was not successful in resolving the QTL, which spanned the 9.5 Mb region (Figure 1).

### Association analysis for AUDPC in the NIL library

**Figure 2:**
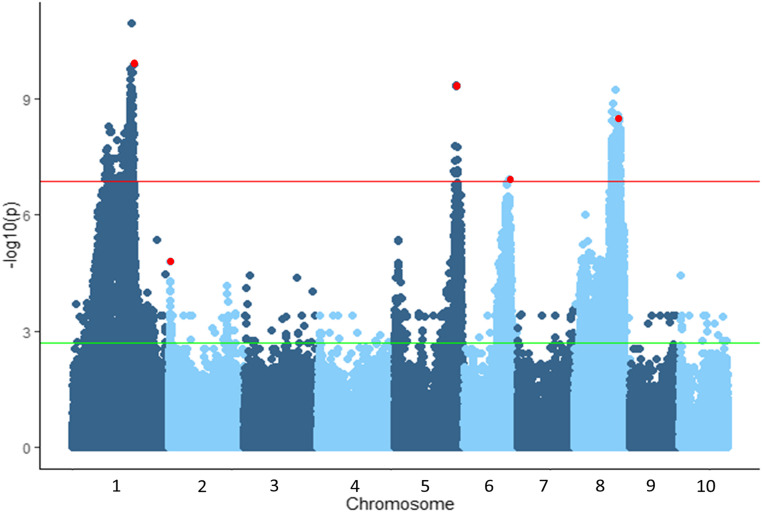
Association analysis of resistance to northern leaf blight in the nested near-isogenic line library using the general linear model in TASSEL. Significance thresholds were determined using the Bonferroni correction factor (red line) and the false discovery rate (green line). The SNPs most significantly associated with resistance by stepwise regression are highlighted in red.

GWAS was employed in the nNIL library with 374,540 genome-wide SNPS (File S1). After Bonferroni correction, four regions of the genome were identified as significantly associated with resistance to NLB (Figure 2), which overlapped known NLB QTL on chromosomes 1, 5, 6 and 8 (Poland *et al.* 2011; Wisser *et al.* 2006). The top three SNPs in each of these significant peaks were chosen to investigate nearby candidate genes (Table 1).

Stepwise regression identified five significant SNPs, including one SNP in each peak, accounting for variation in each peak that was identified based on GWAS as well as an additional SNP on chromosome 2 (Table 1; Table S5). The region spanning the centromere of chromosome 1 (80-200 Mb) had a large introgressions with significant association, reflecting the lack of recombination in NILs covering this region. The most significant SNP identified by GWAS was in a low confidence gene at 1:183,611,752, while the second most significant GWAS association and the most significant SNP identified through stepwise regression at 1:191,522,802, in a gene annotated as a Ca+ transporter. Using GWAS and stepwise regression, the most significant SNP at the QTL on Chromosome 8 was in a gene identified as a protein phosphatase, located at 8:138,561,937. The significant SNP identified through GWAS and stepwise regression on chromosome 6 was located in a gene annotated as encoding an ENTH/VHS/GAT family protein (Table 1).

B73 was the source of the more resistant allele for two of the NLB QTL regions. Two significant SNPs around the qNLB5.05_NAM_ region were identified via GWAS, one of which was located in a gene (GRMZM2G005996) annotated as a mechanosensitive channel of small conductase. Additionally, the SNP located at 5:193,652,476 was identified at the same locus via stepwise regression (Table 1). The second SNP (5:191,129,001) identified via GWAS was located in GRMZM2G112830, a gene annotated as trehalose-6-phosphate phosphatase6. The two genes are separated by approximately 1.3 Mb, substantially reducing the candidate interval for qNLB5.05_NAM_ from a previously reported 9.5 Mb (Poland *et al.* 2011).

The second B73-derived NLB QTL region identified via stepwise regression rested on the short arm of chromosome 2, overlapping with qNLB2.01/2.02_NAM_ (Poland *et al.* 2011). The identified SNP (2:4,263,855) was one of two peaks identified via GWAS in the QTL, but the only one identified as significantly contributing toward resistance. The SNP (2:4,263,855) was located immediately downstream of the *liguleless1* gene (*lg1*, GRMZM2G036297). The second SNP (2:4,847,920) identified via GWAS was located in GRMZM2G032977, annotated as a nuclease PA3. *Liguleless1* was previously identified as a candidate gene via GWAS in the NAM population (Poland *et al.* 2011).

We investigated the NILs that underlie the 5 main peaks that were identified for significance based on GWAS and stepwise regression SNP significance, using the SNP identified via stepwise-regression as the anchor for the five peaks (given that they usually had high GWAS *p*-values). The number of NILs needed to identify these 5 peaks ranged from 8 to 26 NILs, with the indication that the more NILs that spanned the peak, the higher the significance level using GWAS (Table S6). For example, 21 and 26 NILs were spanning the regions at chr 1.06 and 8.05 respectively, whereas only 8 NILs spanned the 2.01/2.02. Comparison of NIL NLB phenotype (*i.e.*, resistant, susceptible and not significantly different from B73) to the direction of allelic effects from founders in the NAM NL JL QTL shows that some of the allele effects are similar. The NILs may harbor additional/different resistance genes for NLB in the same introgression or additional introgressions across the genome. This may be seen in cases where more than one resistance phenotype may be present in 2 or more NILs from the same donor parent.

### liguleless1 is a candidate gene for resistance to NLB

**Figure 3:**
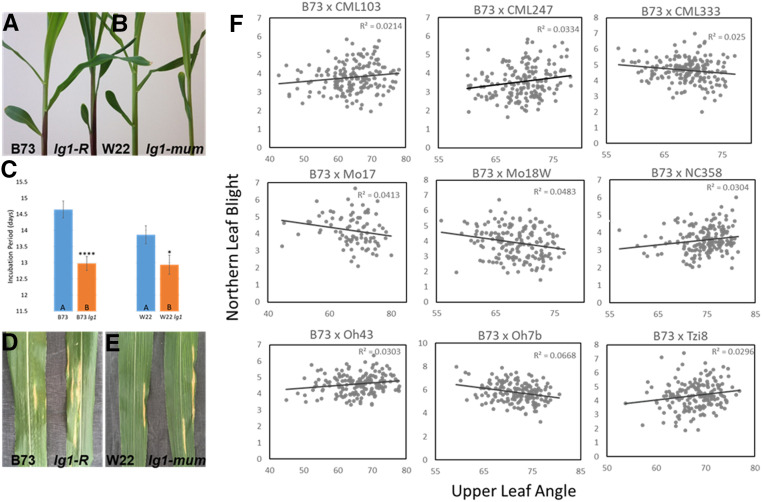
The effect of *liguleless1* (*lg1*) on resistance to northern leaf blight. Leaf architecture is impacted by mutations in *lg1* (A) B73 (left) and *lg1-R* (right), and (B) W22 (left) and *lg1-mum* (right). (C) The *lg1-R* and *lg1-mum* alleles were both significantly more susceptible to northern leaf blight (NLB), measured as incubation period in the greenhouse (the number of days after inoculation that the NLB lesion appeared). Bars represent ± SE. NLB lesions on the leaves of (D) B73 and *lg1-R*, and (E) W22 and *lg1-mum*. Significant positive and negative correlations (F) were identified between the upper leaf angle (Tian *et al.* 2011) and resistance to NLB (NLB index; Poland *et al.* 2011) in 9 RIL populations of the nested association mapping population.

Mutant analysis was conducted to probe the role of the *lg1* gene in the NLB phenotype. Lines carrying two mutant alleles were examined for NLB response in greenhouse trials: the *lg1-R* allele in the B73 background and the *lg1-mum* allele in the W22 background. Incubation period was measured after whorl inoculation with *S. turcica*, which reduced the influence of leaf angle on disease development. Both mutant alleles were significantly more susceptible to NLB compared to respective inbred lines (Figure 3). Incubation period was approximately 1.5 days and 1 day shorter in the *lg1* mutant relative to B73 and W22 inbred lines respectively, indicating increased susceptibility through a more rapid infection cycle in the *lg1* mutants.

In maize, mutations in *lg1* result in more upright leaves (Moreno *et al.* 1997) and natural variation in *lg1* associates with more upright leaves in maize breeding lines (Dzievit *et al.* 2019; Tian *et al.* 2011). A significant negative correlation was identified between middle leaf angle and resistance to NLB (AUDPC) in the 282-line maize diversity panel (Flint-Garcia *et al.* 2005; *P* < 0.001; *R^2^* = 0.175; Figure S6), however, population structure or kinship was not accounted for. At the sub-population level, significant negative correlations between leaf angle and AUDPC were observed in the non-stiff stalk (NSS) and mixed subpopulations (*P* < 0.0001; *R^2^* = 0.285 and *P* = 0.0006; *R^2^* = 0.106 respectively; Figure S6). In these sub-populations, the higher leaf angle (more erect leaves) was correlated with lower levels of disease. Leaf angle and resistance to NLB AUDPC, were not significantly correlated within the stiff stalk (SS), tropical, popcorn or sweet corn sub-populations. GWAS utilizing the 282 maize diversity panel, using the mixed linear model (MLM) to account for population structure and kinship, did not identify a significant association with NLB across *lg1* (Figure S7). To further assess the effect of leaf angle on resistance to NLB, we examined the correlations of upper leaf angle and resistance to NLB index, measured as NLB index (average of three DLA measurements; Poland *et al.*, 2011) in the individual RIL populations of the NAM populations (Tian. *et al.* 2011; Poland *et al.* 2011). Of the 26 RIL populations, five exhibited significant positive correlations between leaf angle and NLB, while four exhibited a significant negative correlations between leaf angle and NLB (Figure 3). The roles of *lg1* in influencing leaf angle and NLB pathogenesis are distinct and putatively multi-allelic.

## Discussion

The genetic architecture of many maize traits have been studied in the NAM population, including flowering time (Buckler *et al.* 2009); leaf architecture (Tian *et al.* 2011); plant height (Peiffer *et al.* 2014); stalk strength (Peiffer *et al.* 2013); resistance to NLB (Poland *et al.* 2011), southern leaf blight (Kump *et al.* 2011), and gray leaf spot (Benson *et al.* 2015); hypersensitive defense response (Olukolu *et al.* 2014); kernel composition (Cook *et al.* 2012); photosynthesis (Wallace *et al.* 2014); inflorescence architecture (Brown *et al.* 2011); and photoperiod sensitivity (Hung *et al.* 2012). We show the power of using a nested NIL library derived from the parents of the NAM population in the validation, phenotypic characterization, fine-mapping, and gene discovery of the QTL for NLB.

### Description and utility of the nNIL library for dissection of QTL

The nNIL library described in this paper is part of a community resource developed by Syngenta as a tool for dissection of important traits (Gandhi *et al.* 2001; Morales *et al.* 2020) and available upon request from the International Maize and Wheat Improvement Center (CIMMYT). We describe a subset of a larger population of NILs derived from 18 donor parents (consisting of founder lines in the NAM population) and backcrossed to B73, that were initially selected to harbor introgressions around known QTL for resistance to NLB. The potential for association mapping in a nNIL population is based on the diverse parentage of the nNILs and the high-density SNP coverage. The library can be used for QTL mapping or confirmation (Morales *et al.* 2020) and individual NILs can be selected for use in fine-mapping of specific loci of interest, to allow testing of candidate genes. The heterozygous introgressions identified in these lines can be exploited for heterogeneous inbred family analysis (Tuinstra *et al.* 1997).

Recombination rates vary across the genome, with increased recombination near the telomeric regions and decreased recombination near the centromeres (Rodgers-Melnick *et al.* 2015). Consequently, introgression sizes identified in this study varied widely and as generally predicted. QTL resolution was poor in regions around centromeres. In a QTL resolution study in Arabidopsis, given similar population resources, RILs provided greater QTL resolution than individual NILs at defining QTL intervals (Keurentjes *et al.* 2007). In this study, we utilized approximately 9% of the number of NILs compared to RILs utilized in the NAM population. The average size of NLB QTL in the NAM RILs was 14.6 Mb (Poland *et al.* 2011), which was smaller than the average introgression size in our NIL library (24.3 Mb), although the range of the introgression sizes was much larger in the nNIL library than compared to that identified through the joint linkage mapping of NLB QTL in the NAM population. The median size of a NAM NLB QTL (9.5 Mb) was, however, much more similar to that of the median NIL introgression size (9.3 Mb). Across the NILs, the range in introgression size varied, with the smallest introgression in the NIL library (59.2 Kb) being smaller than the smallest NAM NLB QTL (855 Kb), and the largest introgression size (188 Mb) being considerably larger than the largest NLB QTL (77.1 Mb). Thus, while the NIL introgressions are generally larger than the average NAM NLB QTL, the range of introgression size is wider, as was also found in the larger set of NILs (Morales *et al.* 2020). Because of the SNP saturation across the genome, we were able to detect introgressions smaller than 5 Mb, which might not have been detectable with a sparser marker dataset. These micro-introgressions do not have the characteristics of sequence artifacts, so are provisionally presumed to be valid.

A NIL library in Arabidopsis detected QTL with smaller allelic effects than those in the corresponding RIL population (Keurentjes *et al.* 2007). In the current study, the mean allelic effect size of QTL detected in the nNIL library (via AUDPC, an area measurement over time from three DLA scores) was similar to that identified in the NAM RIL population (average index of three DLA scores; Poland *et al.* 2011), as suggested by Kaeppler (1997). The range in allelic effects of the nNIL library was approximately three times the size of the allelic effect size of the joint linkage NAM QTL, however care must be given to this interpretation as this maybe a relic of using an average DLA area over time (this study), *vs.* the average DLA scores (Poland *et al.*, 2011). We did not identify additional QTL of smaller effect, apart from those with flowering time effect, in comparison to the set identified in the full NAM population, likely due to the smaller sample size used in this study.

We identified NILs that were either more resistant or more susceptible to NLB than B73. The QTL identified were consistent with those identified in the NAM RIL population. QTL with large effect sizes, associated with donor-derived introgressions, were identified across the centromeric regions of chromosomes 1, 6 and 8. These regions overlapped with the previously described QTL designated as qNLB1.06 and qNLB6.05 (Jamann, *et al.* 2014; Poland *et al.*, 2011). NILs with introgressions across the centromeric region of chromosome 8 overlapped two QTL for resistance to NLB, including qNLB8.06, which overlaps the *Ht2* and *Htn* resistance loci (Chung *et al.* 2010; Hurni *et al.* 2015). Co-localized QTL regions for resistance to NLB between this nNIL study and the larger nNIL library (Morales *et al.* 2020) were identified near qNLB6.05 and in overlapping qNLB8.05 regions. Differences in the disease phenotyping environments, genotyping and analysis methodologies, as well as nNIL library composition maybe have contributed to the limited concordance between the results from the two nNIL libraries. The inoculation of NILs with different *S. turcica* races, will allow development of a “differential series” for major and minor resistance loci for NLB in the B73 background, and differentiate potential race-specificity of NILs harboring qNLB8.06. The introgressions in NILs that span qNLB1.06 and/or qNLB8.06 are large, however, which suggests that lack of recombination will inhibit fine-mapping in those regions, as has been previously observed at qNLB1.06 (Jamann *et al.* 2014).

B73 is considered moderately susceptible to NLB, but known to carry alleles for NLB resistance (Poland *et al.* 2011). In this study, we identified twice as many NILs that were more susceptible to NLB than B73 (lines in which B73-derived resistance was lost with an introgression), than were more resistant to NLB than B73. Additionally, resistance in two of the five QTL regions identified in this study via GWAS and stepwise regression was derived from B73, the recurrent parent of the nNILs: qNLB 2.01 and qNLB5.05. The design of the nNIL library, like the NAM population, was favorable for identifying resistance derived from B73; the nNIL library allowed systematic analysis of the recurrent parent genome for effects on disease, while the donor genomes were only partially represented in this subset of NILs.

A previous study in the 282 maize inbred diversity panel showed a negative genetic correlation between DTA and AUDPC (Wisser *et al.* 2011) that was also observed in the all sub-populations of the 282 maize inbred diversity panel with the exception of the tropical subpopulation. The tropical subpopulation, had less variation for both DTA (showing longer days to flowering) and AUDPC (showing higher levels of resistance). A low negative correlation was detected between DTA and NLB in the nNIL library. This may be due to a small number of NILs with introgressions that were more resistant to NLB and had longer flowering time, either due to linkage (different loci influencing the two traits) or pleiotropy (individual gene[s] influencing both traits). While a significant negative correlation between DTA and resistance to NLB was found in the NAM founder lines, significant negative correlations were found in only 8 of the 26 NAM RIL populations (Poland *et al.* 2011).

The lower correlations between maturity and disease resistance in the RIL and NIL populations indicates that some of the correlations seen across germplasm collections may be due to population structure rather than to linkage and/or pleiotropy (Poland 2010). Despite the overall trend, one outlier was identified. A NIL harboring an introgression that spanned the qNLB1.02 region had increased NLB resistance but reduced days to anthesis relative to B73, confirming a previous report (Jamann *et al*. 2016; Poland *et al*. 2011). Several NILs with longer maturity harbored introgressions at the telomeric end of chromosome 1 that had not been identified previously in NAM QTL mapping. These introgressions either were identified in our study due to an increase in allele effect sensitivity in our population, or because these introgressions co-localized with flowering time effect, which may be been a reduced factor in the NAM QTL mapping where DTA was used as a covariate.

### Association analysis in the nNIL library utilizes ancient recombination for greater QTL resolution

To identify candidate loci or regions for resistance to NLB, we utilized the polymorphisms among the introgression donors at a given locus using association analysis across the nNILs. The uniform B73 genetic background diminished the confounding effect of population structure and kinship. Only a small number of NILs had introgressions in any given genomic region, yet significant effects were identified at 5 regions in the genome, which corresponded with known NLB QTL. Utilizing high density SNP coverage in the ∼400 NILs, we were able to narrow the interval of interest in a region of low recombination (qNLB1.06), identified putative candidate alleles in two other QTL regions of large effect (qNLB8.05, and qNLB6.05), and provided a narrower window of significance for two QTL with B73-derived resistance. One of the B73 QTL, qNLB5.05, was narrowed to two significant peaks about 1 Mb apart, only one of which remained significant via stepwise regression. Likewise, the other B73-derived QTL, qNLB2.01/2.02, harbored two SNPs, approximately 0.6 Mb apart, with the SNP identified via stepwise regression near the *Liguleless*1 gene. While these results appear promising, the SNP coverage, number of overlapping NILs, and the number of donors represented in the NILs have a direct impact on the resolution and interpretation of the findings. B73-derived resistance is likely represented in the anchor genome, and has a higher likelihood of being identified in this germplasm. NILs conferring resistance from donor parents (*i.e.*, non-anchor genome), will need to be further vetted to be sure that the resistance allele is being represented via adequate SNP coverage. Nevertheless, these results do offer a promising way to increase QTL resolution.

### Candidate gene analysis of qNLB2.01 implicates liguleless1

A significant SNP in qNLB2.01/2.02 was identified within ∼600 bp of the *liguleless*1 gene. The resistant allele was derived from B73. NAM GWAS analyses for resistance to NLB had previously identified a significant SNP in the *lg*1 gene (HapMapv1; Poland *et al.* 2011), and within 100 kb of *lg1* (HapMapv2; Chia *et al.* 2012). We used controlled inoculation of *lg1* mutant lines to further probe the role of *lg1* in disease resistance. Because the *lg1* gene influences leaf angle, we chose a disease assay that was intended to minimize the influence of microclimate due to leaf angle or canopy architecture: we applied fungal spores in the whorl and scored the time to primary lesion formation. The two mutants tested were significantly more susceptible to NLB than their wild type counterparts, B73 and W22, with a decrease in incubation period of 1.5 and 1 days, respectively.

The natural variant and mutant alleles of *liguleless1* impact leaf angle and subsequent canopy structure, that ultimately affects plant density (Becraft *et al.* 1990; Sylvester *et al.* 1990; Tian *et al.* 2011; Tian *et al.* 2019). The *lg*1 gene encodes a SQUAMOSA binding protein transactional regulator (Moreno *et al.* 1997). The *lg1* mutants lack the ligule and auricle at the blade/sheath boundary, and have severely upright leaves (Emerson 1912; Becraft *et al.* 1990, Sylvester *et al.* 1990).

A metaQTL was identified on chromosome 2 within a ∼1.05 Mb region that was associated with both leaf angle and resistance to maize rough dwarf virus disease (Wang *et al.* 2016). It was postulated that *lg1* might be a co-contributor to both the leaf angle (as shown in Tian *et al.* 2011; Li *et al.* 2015) and resistance to maize rough dwarf disease (Wang *et al.* 2016). The expression of *lg1* in leaf tissue (Stelpflug *et al.* 2016) and the implications of the role of *lg1* in disease resistance found in our study are consistent with a pleiotropic role for this gene. The *Lg1* gene was implicated through GWAS in the nNIL and NAM populations (Poland *et al.* 2011; Chia *et al.* 2012), both of which feature B73 as genetic background (approximately 97% and 50%, respectively).

The increases in yield of maize production achieved over the last 50 years has been derived through increased planting population densities, and the indirect selection of corresponding adaptive traits, such as leaf angle (Duvick 2005) and stress tolerance (Tollenaar and Wu 1999). In rice, an increase in methyl jasmonate was found to decrease leaf angle by inhibiting brassinosteroid (BR) biosynthesis, the BR signaling pathway, and BR-induced gene expression (Gan *et al.* 2015). Auxin and BR signaling pathways were associated with leaf angle, using the *Lg1* homolog in wheat, TsaSPL8 (Liu *et al.* 2019). A plant architecture QTL in maize was recently dissected (Tian *et al.* 2019) that regulates BR and leaf angle, increasing yield in dense population environments, and demonstrated the involvement of LG1 in the regulation of genes controlling these traits. The *sympathy for the ligule* gene, a modifier of the *liguleless narrow1* gene, was also shown to have a pleiotropic role in maize leaf architecture and disease resistance (Anderson *et al.* 2019). The observation of both positive and negative correlations between leaf angle and NLB (AUDPC) in the five and four RIL families of the NAM population, respectively, suggests that *lg1* may be multi-allelic, may have a role in leaves that is distinct from its role in leaf angle, and that *lg1* influences pathogenesis.

The nNIL library characterized in this study is a valuable resource for the genetic characterization and dissection of important traits. The GBS sequencing data for this population allows for highly resolved introgression breakpoints. As with other NIL resources, the population can be utilized to identify and confirm QTL in maize and individual NILs can be used for use in fine-mapping QTL, and for detailed studies on the morphological and physiological mechanisms associated with them. A unique feature of this multi-parental NIL population is its utility for association mapping. Indeed, polymorphisms at *lg1* were identified by association with NLB in this population, confirming the association previously observed in the NAM population by GWAS. The nNIL library can be utilized as a useful resource to further refine traits that have been previously studied in the maize NAM population, or are yet to be characterized.
